# Enlarged adipocytes from subcutaneous vs. visceral adipose tissue differentially contribute to metabolic dysfunction and atherogenic risk of patients with obesity

**DOI:** 10.1038/s41598-021-81289-2

**Published:** 2021-01-19

**Authors:** Juan Antonio Suárez-Cuenca, Gustavo De La Peña-Sosa, Karen De La Vega-Moreno, Diana Zaineff Banderas-Lares, Moisés Salamanca-García, José Enrique Martínez-Hernández, Eduardo Vera-Gómez, Alejandro Hernández-Patricio, Carlos Ramiro Zamora-Alemán, Gabriela Alexandra Domínguez-Pérez, Atzín Suá Ruíz-Hernández, Juan Ariel Gutiérrez-Buendía, Alberto Melchor-López, Moisés Ortíz-Fernández, Jesús Montoya-Ramírez, Omar Felipe Gaytán-Fuentes, Angélica Toríz-Ortíz, Mario Osorio-Valero, Julita Orozco-Vázquez, Sofía Lizeth Alcaráz-Estrada, Martha Eunice Rodríguez-Arellano, Brenda Maldonado-Arriaga, Rebeca Pérez-Cabeza de Vaca, Mónica Escamilla-Tilch, Juan Antonio Pineda-Juárez, Mario Antonio Téllez-González, Silvia García, Paul Mondragón-Terán

**Affiliations:** 1 Laboratory of Experimental Metabolism and Clinical Research, Division of Research, Department of Clinical Research, Centro Médico Nacional “20 de Noviembre”, ISSSTE, 502, San Lorenzo, Colonia Del Valle, Delegación Benito Juárez, 03100 Mexico City, Mexico; 2 Internal Medicine Department, H.G.Z. No. 58 “Manuel Ávila Camacho”, IMSS, and Hospital General “Xoco” SS CDMX, 03340 Mexico City, Mexico; 3 Pathology Department, Centro Médico Nacional “20 de Noviembre”, ISSSTE, 03104 Mexico City, Mexico; 4 Internal Medicine Department, H.G.Z. No. 8 “Gilberto Flores Izquierdo”, IMSS and Hospital General “Xoco” SS CDMX, 03340 Mexico City, Mexico; 5 Bariatric Surgery Department, Centro Médico Nacional “20 de Noviembre”, ISSSTE, 03100 Mexico City, Mexico; 6 Diagnostic Imaging Department, Centro Médico Nacional “20 de Noviembre”, ISSSTE, 03100 Mexico City, Mexico; 7 Laboratorio de Medicina Genómica, Centro Médico Nacional “20 de Noviembre”, ISSSTE, 03100 Mexico City, Mexico; 8 Laboratorio de Medicina Genómica, Hospital Regional “Lic, Adolfo López Mateos”, ISSSTE, Mexico City, Mexico; 9 Coordination of Research and Tissue Engineering & Regenerative Medicine Research Group, Centro Médico Nacional “20 de Noviembre”, ISSSTE, 03100 Mexico City, Mexico

**Keywords:** Biochemistry, Cell biology, Physiology, Biomarkers, Cardiology, Endocrinology, Medical research, Pathogenesis, Risk factors

## Abstract

Morphological characteristics and source of adipose tissue as well as adipokines may increase cardiometabolic risk. This study aimed to explore whether adipose tissue characteristics may impact metabolic and atherogenic risks. Subcutaneous Adipose Tissue (SAT), Visceral Adipose Tissue (VAT) and peripheral blood were obtained from obese patients submitted to bariatric surgery. Adipose tissue (morphometry), plasma adiponectin, TNF-α, resistin (multiplexing) and biochemical chemistry were analyzed; as well as endothelial dysfunction (Flow Mediated Dilation, FMD) and atherogenesis (Carotid Intima Media Thickness, CIMT). Subgroups divided by adipocyte size and source were compared; as well as correlation and multivariate analysis. Sixty patients 36.6% males, aged 44 years-old, BMI 46.7 kg/m^2^ were included. SAT’s adipocytes showed a lower range of size expandability than VAT’s adipocytes. Independent from their source, larger adipocytes were associated with higher glucose, lower adiponectin and higher CIMT. Particularly, larger adipocytes from SAT were associated with higher blood pressure, lower insulin and HDL-cholesterol; and showed positive correlation with glucose, Hb_A1c_, systolic/diastolic values, and negatively correlated with insulin and adiponectin. VAT’s larger adipocytes particularly associated with lower resistin and lower FMD values. Gender and Diabetes Mellitus significantly impacted the relation of adipocyte size/source with the metabolic and atherogenic risk. Multivariable analysis suggested hypertension-resistin-Hb_A1c_ interactions associated with SAT’s larger adipocytes; whereas potential insulin-adiponectin associations were observed for VAT’s larger adipocytes. Adipocyte morphology and source are differentially related with cardiometabolic and atherogenic risk in population with obesity, which are potentially affected by gender and Diabetes Mellitus.

## Introduction

Overweight and obesity show an increasing prevalence in Western countries^1^ which is relevant due to the relation with co-morbidities such as cardiovascular risk (CVR), apnea–hypopnea syndrome, type 2 Diabetes Mellitus (t2DM), non-alcoholic fatty liver disease and certain types of cancer^2,3^.

The precise mechanisms underlying organ damage in subjects with obesity have not been fully described. Nevertheless, obesity’s CVR has been related to low grade inflammation and subclinical vascular damage. In the other hand, adipose tissue represents a good candidate with potential mechanisms for a key role during CVR in obesity. Adipose tissue constitutes the main site of storage for excessive energy derived from food intake^4,5^, and nowadays, adipose tissue is recognized as an endocrine organ, which secretes many peptide hormones and cytokines “adipokines”, owning pro-inflammatory and pro-atherogenic properties^6–8^. Likewise, visceral adipose tissue (VAT) has been characterized as a predominantly pro-inflammatory tissue; and has been more associated with a higher risk for t2DM, hypertension and dyslipidemia than subcutaneous adipose tissue (SAT)^5,9–11^.

Adipocyte remodeling has been related with specific endocrine and cardiometabolic profiles^5,8,12^. Experimental data from murine models suggest that adipose tissue expandability and/or adipocyte size are associated with insulin resistance, even in the absence of pro-inflammatory conditions^9^, whereas adipose tissue cell size, lipid turnover and adipocytes differentiation have been shown to affect metabolic function and CVR^13,14^. Similar findings regarding morphological/metabolic characteristics of adipose tissue and their relation with cardiometabolic disease have been observed in human studies^15–20^.

Nevertheless, controversial observations about the metabolic meaning of adipose tissue morphology have raised some questions; such as whether adipocyte´s size might predict metabolic health, or how small *vs* large adipocytes differentially contribute to metabolic dysfunction. Enlarged adipocytes are usually related to metabolic dysfunction, whereas small adipocytes are associated with metabolic benefits^15,16,20^; however, paradoxical data have been reported in murine models: (1) adipocyte enlargement observed in SNTB2^−/−^ deficient mice correlates with post-prandial reduced serum glucose, glycerol and lower hepatic triglycerides^21^; while adipocyte hypertrophy in both, SNTB2^−/−^ and Collagen 6 deficient mice enables a normal or improved insulin sensitivity^21,22^; (2) small adipocytes described in the Xylt2^−/−^ deficient lipodystrophic mice associates with a metabolic profile of glucose intolerance, insulin resistance and increased serum triglycerides, as well as inflammatory infiltration of adipose tissue and higher expression of pro-inflammatory mediators^23^. Similarly, increased proportion of small adipocytes in mesenteric fat tissue is related to increased body weight, higher fat mass and decreased insulin sensitivity in the mice model of letrozole-induced polycystic ovary syndrome^24^; and (3) non-significant effect of adipocyte size on metabolic dysfunction has been suggested by data from murine DLK1-induced decrease in adipocyte size^25^. Some of these controversial observations have been replicated in human studies: (1) very small adipocytes have been associated with metabolic dysfunction, reflected by impaired adipogenesis, increased expression of pro-inflammatory cytokines and decreased expression of genes that regulate adipose tissue fatty acid storage^26–28^; (2) small SAT adipocytes are predominant in subjects during overfeeding^29^, and subjects with adipose distribution of metabolic risk^30,31^; and (3) lack of association between the diameter of larger adipocytes with insulin-resistance^26^. In fact, adipocyte size resulted lower predictive of metabolic risk profile than fat mass and body fat distribution in a study cohort^32^.

These controversial findings regarding metabolic implications of adipocyte size suggest that there could be additional factors potentially interacting, where adipose tissue source, adipokines and/or cell differentiation may play a critical role for metabolic dysfunction and CVR^25,33,34^.

Whether pathophysiological mechanisms including adipose mass expandability, adipocyte hypertrophy, subcutaneous *vs* visceral source of adipocytes and/or adipokine production, are related to CVR remain unclear. A comprehensive characterization of the adipose tissue morphology, fat tissue source and adipokines may be useful for better understanding of the role of adipose tissue in metabolic health/dysfunction and CVR. Therefore, the present study aimed to explore the role of morphological-biochemical as well as the source of adipocyte, impact on the metabolic and atherogenic risk in population with obesity.

## Results

The study population was constituted by 60 patients with obesity, mean aged 44 years old, 36.6% male, mean BMI 46.7 kg/m^2^, and two thirds of them met criteria for metabolic syndrome (MS) according to NCEP/ATP III, as well as biochemical evidence of insulin resistance evidenced by plasma glucose, Hb_A1c_ and HOMA-IR (Table 1).

**Table 1:** Study population (n = 60).

Age (years old)	44.2 ± 9.5
Female n (%)	38 (63)
Weight (kg)	124.0 ± 22.3
Height (m)	1.63 ± 0.07
BMI (kg/m^2^)	46.7 ± 8.6
Waist circumference (cm)	135.3 ± 15.8
Systolic blood pressure (mmHg)	123.8 ± 13.4
Diastolic blood pressure (mmHg)	79.4 ± 9.8
Glucose (mg/dL)	106.8 ± 24.0
Hb_A1c_ (%)	5.9 ± 0.6
Insulin (mUI/mL)	33.7 ± 26.2
HOMA-IR	8.9 ± 7.7
Total Cholesterol (mg/dL)	175.9 ± 39.1
HDLc (mg/dL)	44.1 ± 13.4
LDLc (mg/dL)	109.5 ± 33.4
Tryglicerides (mg/dL)	138.7 ± 60.2
TNFα (pg/mL)	52.2 ± 74.3
Resistin (pg/mL)	6862 ± 1605
Adiponectin (pg/mL)	25.9 ± 5.2

Continuous variables are presented as mean ± SD, and categorical variables as n (%).

BMI, body mass index; Hb_A1c_, glycated hemoglobin; LDLc, Low density lipoproteins cholesterol; HDLc, High density lipoproteins cholesterol; TNF α, tumor necrosis factor α.

### Adipocyte size/source and cardiometabolic risk factors

**Figure 1:**
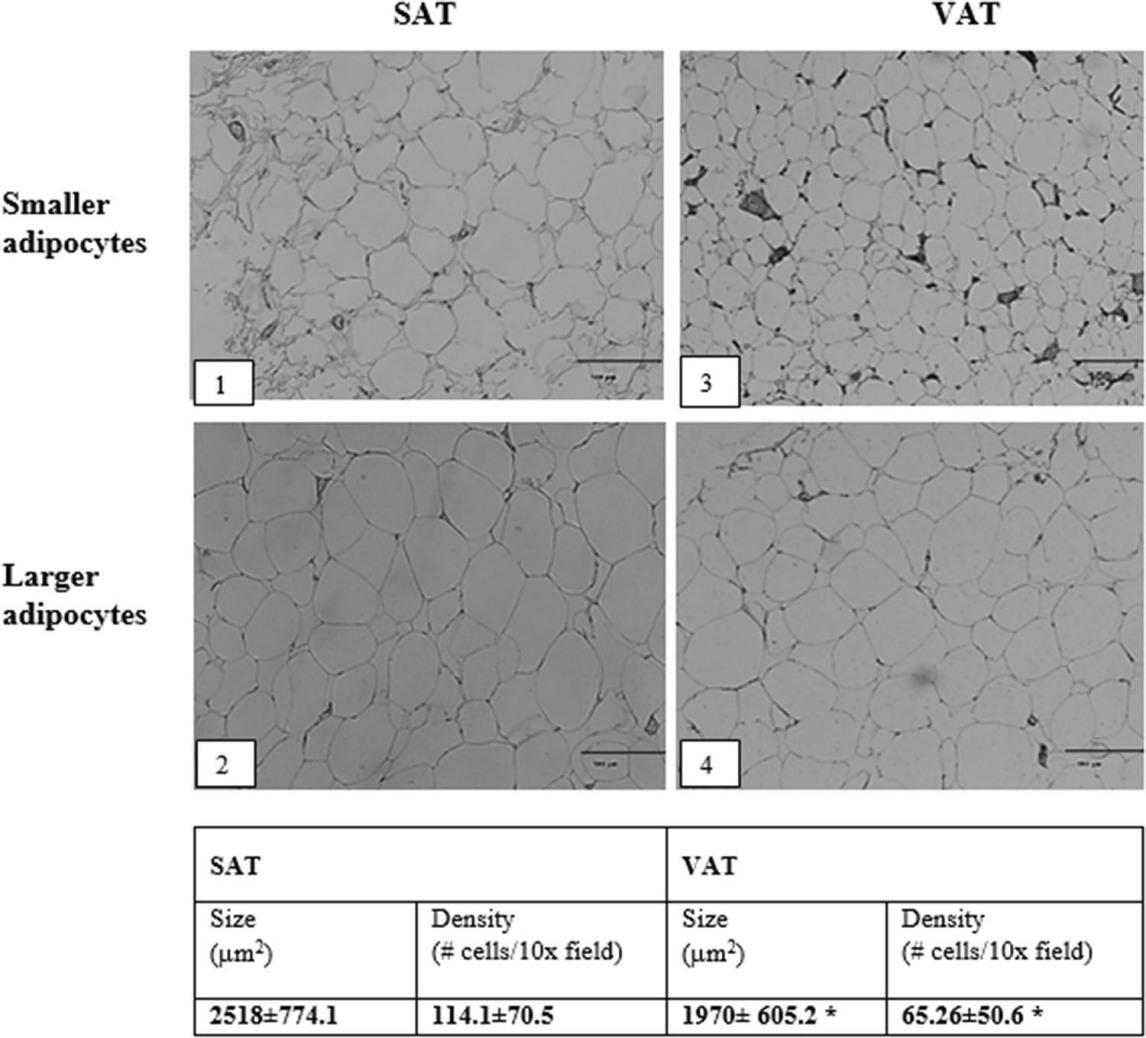
Characterization of adipose tissue (adipocytes size and density). The figure shows a representative image of SAT, including smaller adipocytes (1) and larger adipocytes (2) as well as representative image of VAT including smaller adipocytes (3) and larger adipocytes (4). Results are shown as mean ± standard deviation. SAT, subcutaneous adipose tissue; VAT, visceral adipose tissue. (*) = statistical difference p < 0.05, one-tail, T-test. Images in the figure were analyzed with the software Image J. (v. 1.53g). URL: https://imagej.nih.gov/ij.

Morphological characteristics of adipocytes, obtained either from subcutaneous or visceral source, were determined. We observed significant difference in the size and density of adipocytes from both types of adipose tissues, with a lower range of cell size in SAT (Fig. 1).

In order to evaluate the relation of adipocyte’s characteristics with the cardiometabolic risk, the study population was divided according to adipocyte´s size median value, either from SAT or VAT, as shown in the Tables 2 and 3, respectively. Subsequent analyses by gender and t2DM were performed due to potential metabolic influence.

**Table 2:** SAT’S adipocyte size and clinical-biochemical characteristics (n = 60).

	Smaller adipocytes (n = 30)					Larger adipocytes (n = 30)				
	All	By gender		By t2DM		All	By gender		By t2DM	
		Female	Male	Non t2DM	t2DM		Female	Male	Non t2DM	t2DM
Age (years)	41.3 ± 12.1	45.3 ± 10.1	37.4 ± 9.0	45.5 ± 8.1	42.1 ± 11.5	41.6 ± 7.7	43.9 ± 8.6	47.4 ± 9.3^g^	44.0 ± 10.0	46.0 ± 7.9
Weight (kg)	121.2 ± 23.0	121.7 ± 21.2	119.7 ± 30.3	121.8 ± 30.4	120.8 ± 30.4	128.1 ± 21.3	128.8 ± 20.3	126.8 ± 24.3	130.3 ± 26.8	126.2 ± 15.7
Height (m)	1.65 ± 0.09	1.61 ± 0.09	1.62 ± 0.07	1.64 ± 0.1	1.59 ± 0.07	1.63 ± 0.1	1.68 ± 0.07^g^	1.61 ± 0.04	1.65 ± 0.06	1.66 ± 0.07^DM^
BMI (kg/m^2^)	46.5 ± 8.1	46.7 ± 7.1	45.8 ± 11.4	44.9 ± 10.8	47.6 ± 5.7	47.0 ± 9.3	46.0 ± 8.7	49.1 ± 10.5	47.1 ± 10.0	46.2 ± 8.9
Waist circumference (cm)	134.8 ± 14.1	133.7 ± 11.0	138.3 ± 22.3	129.0 ± 8.0	138.7 ± 16.0	135.8 ± 17.6	137.7 ± 19.3	132.1 ± 13.7	135.6 ± 17.1	135.9 ± 18.6
SBP (mmHg)	118.8 ± 15.6	123.3 ± 16.0	114.9 ± 11.2	118.8 ± 10.0	123.0 ± 18.2	128.1 ± 11.0*	126.1 ± 10.4	126.4 ± 12.5	124.2 ± 9.6	127.9 ± 12.0
DBP (mmHg)	76.7 ± 9.4	77.9 ± 10.0	73.1 ± 6.9	77.5 ± 8.7	76.2 ± 10.2	82.1 ± 9.4*	81.7 ± 9.2	82.8 ± 9.8^g^	81.0 ± 8.2	83.0 ± 10.2
**Adipokines and CHs**										
TNF-α (pg/mL)	53.6 ± 78.0	53.5 ± 83.8	48.8 ± 57.4	60.9 ± 79.8	46.4 ± 76.7	50.9 ± 72.1	40.2 ± 64.3	73.5 ± 86.4	47.4 ± 63.6	56.1 ± 82.6
Resistin (pg/mL)	6866 ± 1327	7012 ± 800	6427 ± 2343	6678 ± 1895	6988 ± 829	6846 ± 1828	7092 ± 1883	6428 ± 2031	6844 ± 2080	6848 ± 1855
Adiponectin (pg/mL)	28.2 ± 4.6	27.6 ± 4.8	25.7 ± 6.7	26.8 ± 5.6	27.4 ± 5.1	24.4 ± 5.3*	24.1 ± 5.5^g^	26.7 ± 3.8	24.9 ± 5.2	24.9 ± 5.2
Glucose (mg/dL)	96.9 ± 10.7	110.5 ± 26.3	97.0 ± 8.8	91.6 ± 4.7	117.8 ± 26.0	117.3 ± 29.5*	105.6 ± 19.1	107.5 ± 33.9	92.5 ± 5.3	118.3 ± 28.3
Insulin (mUI/mL)	42.4 ± 31.1	45.8 ± 36.4	24.6 ± 11.2	30.0 ± 19.4	47.5 ± 38.9	27.3 ± 15.4*	23.1 ± 10.2^g^	36.8 ± 21.0	28.3 ± 17.1	26.3 ± 14.1
Hb_A1c_ (%)	5.9 ± 0.5	6.1 ± 0.6	5.7 ± 0.4	5.7 ± 0.3	6.3 ± 0.5	6.1 ± 0.7	5.8 ± 0.5	6.1 ± 0.7	5.5 ± 0.2	6.3 ± 0.6
HOMA-IR	10.9 ± 9.9	12.9 ± 11.2	6.1 ± 3.0	6.2 ± 4.6	13.8 ± 11.3	7.1 ± 4.2	5.8 ± 2.5^g^	10.0 ± 5.9	6.5 ± 3.9	7.7 ± 4.5
Antidiabetic drug n (%)	19 (63.3)	14 (60.8)	4 (57.1)	8 (66.6)	10 (55.5)	13 (43.3)	7 (35.0)	3 (30.0)	5 (35.7)	5 (31.2)
Total cholesterol (mg/dL)	180.6 ± 35.4	178.3 ± 44.6	179.0 ± 38.7	183.6 ± 42.7	175.0 ± 43.5	175.5 ± 35.0	183.6 ± 33.9	159.2 ± 33.9	174.7 ± 40.2	176.1 ± 31.7
HDL cholesterol (mg/dL)	50.4 ± 14.0	42.2 ± 13.7	49.5 ± 17.3	53.9 ± 17.0	36.9 ± 7.2	41.7 ± 7.7*	46.2 ± 12.7	40.3 ± 9.7	45.1 ± 11.8	43.5 ± 12.4
LDL cholesterol (mg/dL)	108.7 ± 34.9	112.2 ± 33.0	101.7 ± 41.4	107.8 ± 34.8	110.9 ± 35.6	110.3 ± 32.5	115.8 ± 36.5	101.5 ± 25.2	109.2 ± 32.1	112.7 ± 35.6
Triglycerides (mg/dL)	140.1 ± 55.9	139.0 ± 57.7	151.9 ± 52.7	138.3 ± 52.1	144.4 ± 59.5	135.3 ± 65.1	139.2 ± 71.5	126.8 ± 50.6	126.0 ± 58.4	142.9 ± 71.0

Continuous variables presented as mean ± standard deviation and categorical variables as n (%).

SAT, subcutaneous adipose tissue; t2DM, type 2 diabetes mellitus (includes cases of impaired fasting glucose); BMI, body mass index; SBP, systolic blood pressure; DBP, diastolic blood pressure; CHs, carbohydrates; Hb_A1c_, glycosylated hemoglobin.

Mean comparison was performed by one-tail, T-test or U-Mann Whitney as appropriate; whereas categorical data were compared by Fisher exact test. (*) indicates p < 0.05, mean difference between smaller vs. larger adipocytes; (g) indicates p < 0.05, mean difference regarding adipocyte`s size, subgrouped by gender; (DM) indicates p < 0.05, mean difference regarding adipocyte’s size, subgrouped by presence of t2DM (includes cases of impaired fasting glucose).

**Table 3:** VAT’S adipocyte size and clinical-biochemical characteristics (n = 60).

	Smaller adipocytes (n = 30)					Larger adipocytes (n = 30)				
	All	By gender		By t2DM		All	By gender		By t2DM	
		Female	Male	Non t2DM	t2DM		Female	Male	Non t2DM	t2DM
Age (years)	41.6 ± 11.8	44.4 ± 9.9	38.5 ± 11.5	45.9 ± 8.4	41.4 ± 11.2	41.4 ± 8.5	44.9 ± 8.8	45.9 ± 8.9	43.6 ± 9.7	46.7 ± 7.8
Weight (kg)	128.3 ± 21.2	128.4 ± 19.8	128.0 ± 28.2	136.4 ± 22.8	122.9 ± 18.7	121. ± 23.1	120.7 ± 21.9	121.6 ± 26.2	117.7 ± 30.4	123.9 ± 14.7
Height (m)	1.65 ± 0.10	1.65 ± 0.09	1.59 ± 0.04	1.66 ± 0.08	1.62 ± 0.09	1.64 ± 0.08	1.63 ± 0.07	1.62 ± 0.06	1.63 ± 0.08	1.63 ± 0.06
BMI (kg/m^2^)	48.0 ± 8.6	47.4 ± 8.1	50.5 ± 10.5	49.6 ± 9.0	47.0 ± 8.3	45.5 ± 8.7	45.1 ± 7.4	46.2 ± 10.9	44.0 ± 10.9	46.8 ± 6.2
Waist circumference (cm)	136.9 ± 16.1	135.5 ± 14.5	142.7 ± 22.1	132.8 ± 10.3	139.7 ± 18.9	133.7 ± 15.6	135.6 ± 16.8	130.3 ± 13.4	132.4 ± 16.7	134.8 ± 15.1
SBP (mmHg)	122.4 ± 14.3	123.5 ± 14.8	118.0 ± 12.1	120.3 ± 8.3	123.8 ± 17.3	125.3 ± 12.6	126.1 ± 11.9	123.6 ± 13.6	122.9 ± 11.4	127.3 ± 13.3
DBP (mmHg)	78.4 ± 10.9	79.1 ± 11.3	75.7 ± 9.6	77.0 ± 9.0	79.3 ± 12.2	80.2 ± 8.2	80.5 ± 7.2	80.5 ± 9.9	81.4 ± 7.7	79.7 ± 8.7
**Adipokines and CHs**										
TNF-α (pg/mL)	54.6 ± 75.2	43.1 ± 66.4	95.0 ± 96.3	42.8 ± 61.0	62.7 ± 84.6	49.8 ± 74.6	52.3 ± 85.2	46.1 ± 58.2	62.7 ± 78.4	38.7 ± 72.0
Resistin (pg/mL)	7305 ± 1017	7306 ± 1102	7303 ± 721	7583 ± 925	7114 ± 1061	6423 ± 1987*	6729 ± 1624	5950 ± 2457	6078 ± 2344	6722 ± 1643
Adiponectin (pg/mL)	27.8 ± 4.5	27.6 ± 4.3	28.3 ± 5.6	27.7 ± 4.9	27.9 ± 4.4	23.7 ± 5.2*	23.0 ± 5.7^g^	24.9 ± 4.2	24.2 ± 5.3	23.1 ± 5.3^DM^
Glucose (mg/dL)	99.9 ± 20.9	104.0 ± 11.4	98.7 ± 10.3	92.8 ± 5.2	109.6 ± 8.8	111.1 ± 25.5*	113.6 ± 32.0	105.6 ± 32.5	91.4 ± 4.8	127.5 ± 36^DM^
Insulin (mUI/mL)	36.5 ± 30.5	38.2 ± 34.3	30.3 ± 6.7	29.9 ± 11.0	40.8 ± 38.0	30.4 ± 20.0	29.6 ± 18.9	31.7 ± 22.8	28.3 ± 22.4	32.8 ± 17.5
Hb_A1c_ (%)	5.9 ± 0.5	6.1 ± 0.6	5.7 ± 0.4	5.6 ± 0.3	6.3 ± 0.5	6.3 ± 0.7	5.8 ± 0.5	6.1 ± 0.6	5.6 ± 0.3	6.3 ± 0.6
HOMA-IR	9.6 ± 9.3	10.2 ± 10.5	7.5 ± 2.3	6.2 ± 2.3	11.4 ± 11.1	8.2 ± 5.5	8.0 ± 5.2	8.6 ± 6.3	6.5 ± 5.1	10.3 ± 5.5
Antidiabetic drug n(%)	17 (56.6)	13 (54.2)	3 (50.0)	7 (58.3)	9 (50.0)	15 (50.0)	8 (42.1)	4 (36.4)	6 (42.8)	6 (37.5)
Total cholesterol (mg/dL)	172.9 ± 37.0	171.5 ± 39.1	178.8 ± 29.2	176.1 ± 36.5	170.8 ± 38.3	179.0 ± 41.6	192.4 ± 38.0	161.1 ± 39.3	181.1 ± 45.3	180.8 ± 37.9
HDL cholesterol (mg/dL)	46.2 ± 12.8	42.7 ± 11.7	53.9 ± 15.4	50.6 ± 14.4	41.2 ± 10.9	41.4 ± 10.3	45.9 ± 15.2	38.7 ± 9.5^g^	47.9 ± 15.6	38.8 ± 10.1
LDL cholesterol (mg/dL)	101.1 ± 26.6	103.0 ± 32.3	101.6 ± 32.2	102.4 ± 29.2	102.9 ± 34.1	113.9 ± 33.2	128.4 ± 32.3^g^	101.6 ± 32.9	113.8 ± 35.7	122.4 ± 34.2
Triglycerides (mg/dL)	153.8 ± 59.0	146.3 ± 73.5	149.3 ± 52.2	134.4 ± 55.3	154.7 ± 76.3	169.9 ± 59.8	130.3 ± 50.3	130.8 ± 52.4	129.3 ± 56.4	131.4 ± 46.3

Continuous variables presented as mean ± standard deviation and categorical variables as n (%).

VAT, visceral adipose tissue; t2DM, type 2 diabetes mellitus (includes cases of impaired fasting glucose); BMI, body mass index; SBP, systolic blood pressure; DBP, diastolic blood pressure; CHs, carbohydrates; Hb_A1c_, glycosylated hemoglobin.

Mean comparison was performed by one-tail, T-test or U-Mann Whitney as appropriate; whereas categorical data were compared by Fisher exact test. (*) indicates p < 0.05, mean difference between smaller vs. larger adipocytes; (g) indicates p < 0.05, mean difference regarding adipocyte`s size, subgrouped by gender; (DM) indicates p < 0.05, mean difference regarding adipocyte`s size, subgrouped by presence of t2DM (includes cases of impaired fasting glucose).

Larger adipocytes were related to higher plasma glucose and lower adiponectin, independently from their source SAT or VAT. Larger SAT’s adipocytes were particularly related with higher blood pressure, as well as lower HDL-cholesterol, lower plasma insulin and trend to lower HOMA-IR. On the other hand, larger VAT’s adipocytes were related with lower plasma resistin. Both, gender and t2DM modified the effect of larger adipocytes. Gender modified the effect of adipocytes size on a higher amount of variables (in SAT larger adipocytes: diastolic blood pressure, adiponectin, insulin and HOMA-IR; while in VAT larger adipocytes: adiponectin, HDL cholesterol and LDL cholesterol). On the other hand, t2DM impacted the adipocyte’s effect on fewer variables, mainly in VAT (in VAT larger adipocytes: resistin and adiponectin).

**Figure 2:**
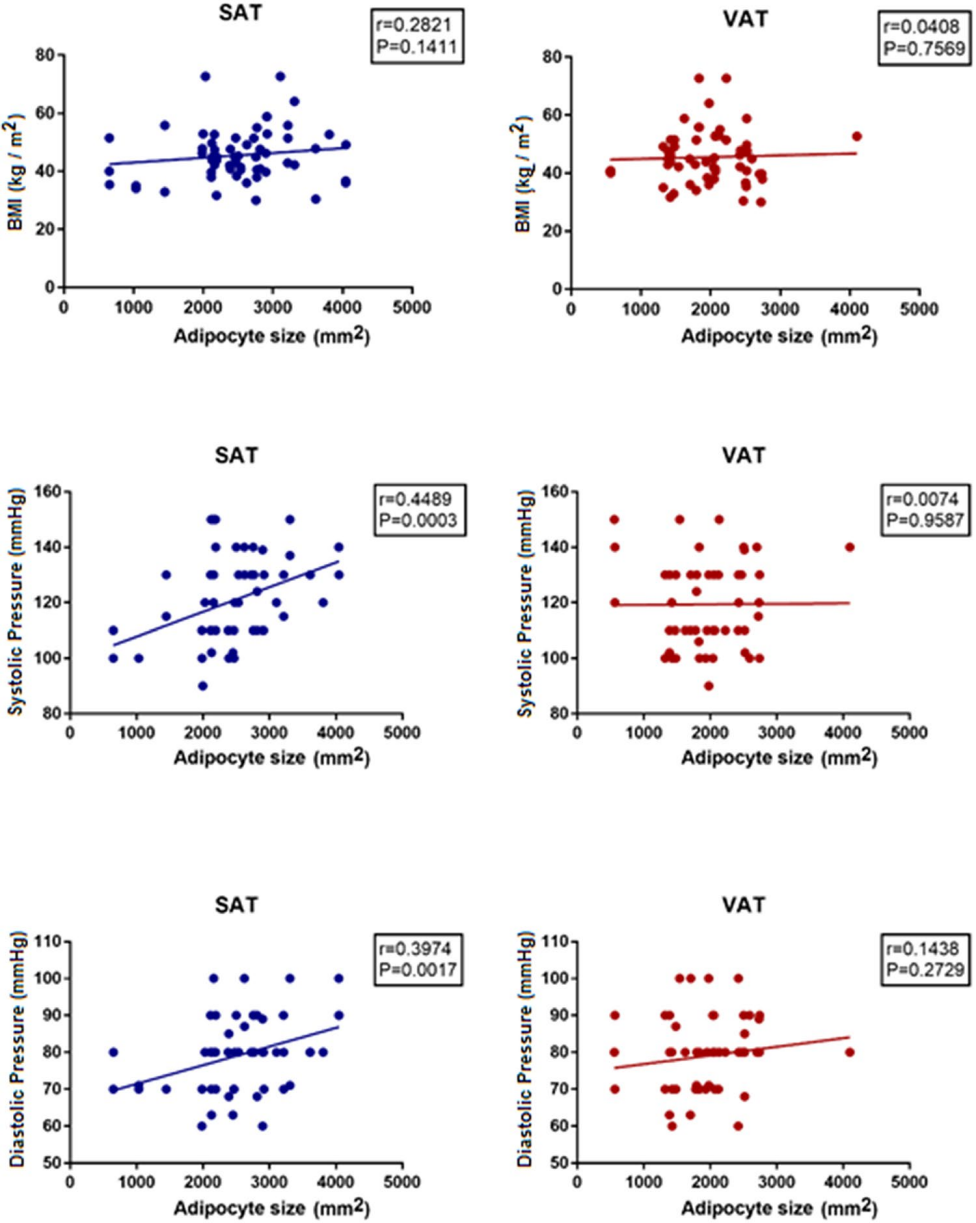
Adipocyte size, BMI and blood pressure. The graphs show the correlation between adipocyte’s sizes in VAT and SAT with BMI, systolic and diastolic pressure. SAT, subcutaneous adipose tissue; VAT, visceral adipose tissue. Figure was produced with the software Graph Pad Prism 7.0. URL https://graphpad-prism.software.informer.com/7.0.

Further characterization of the relation between adipocyte size with cardiometabolic risk markers showed that adipocyte size particularly from SAT (SAT[as]), but not adipocyte size from VAT (VAT[as]), positively correlated with systolic and diastolic blood pressures (Fig. 2).

**Figure 3:**
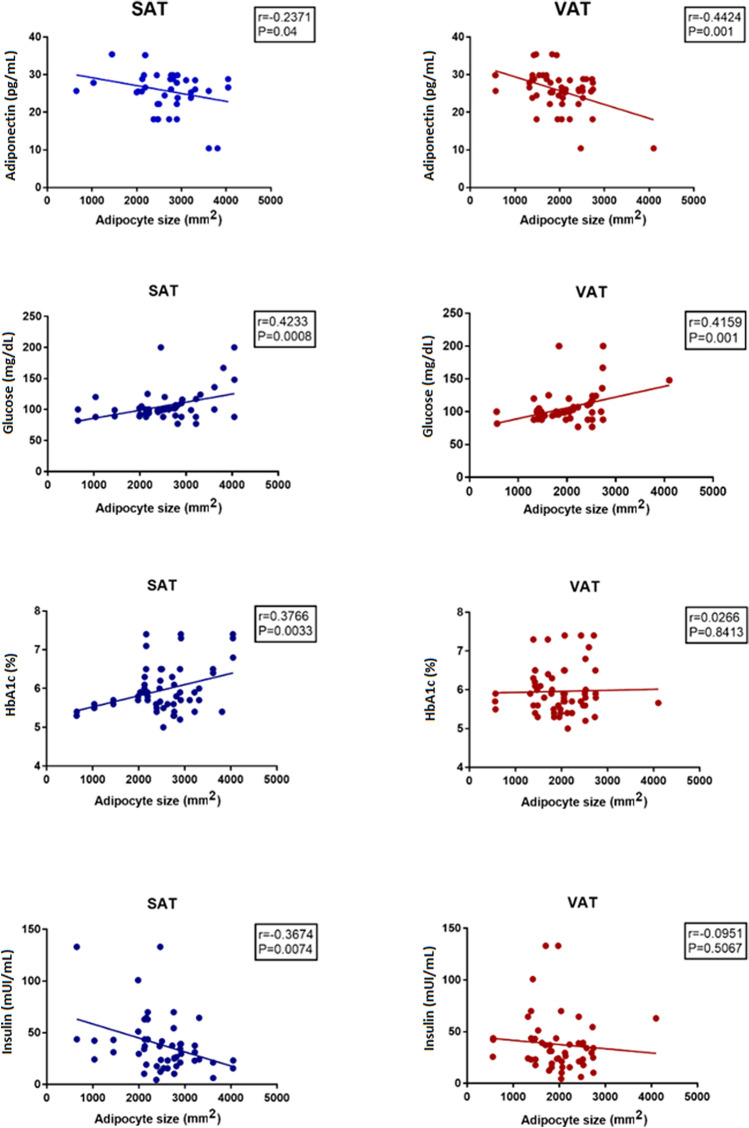
Adipocyte size, adiponectin and glucose metabolism. The graphs show the correlation between adipocyte’s size in VAT and SAT with adiponectin and glucose metabolism factors. SAT, subcutaneous adipose tissue; VAT, visceral adipose tissue; Hb_A1c_, glycosylated hemoglobin. Figure was produced with the software Graph Pad Prism 7.0. URL https://graphpad-prism.software.informer.com/7.0.

Regarding metabolic profile, adipocyte size was related again with plasma insulin, adiponectin (negative correlations) as well as with blood glucose and Hb_A1c_ (positive correlations) (Fig. 3).

In addition, adipocyte size showed general correlation with triglycerides (SAT[as] r = 0.31; p = 0.01, VAT[as] r = 0.32; p = 0.01) and HDL-cholesterol (SAT[as] r = 0.35; p = 0.006, VAT[as] r = 0.32; p = 0.01), but did not correlate with LDL-cholesterol (SAT[as] r = 0.11; p = 0.38, VAT[as] r = 0.20; p = 0.12).

### Adipocyte’s size/source, endothelial dysfunction and atherogenic risk

**Figure 4:**
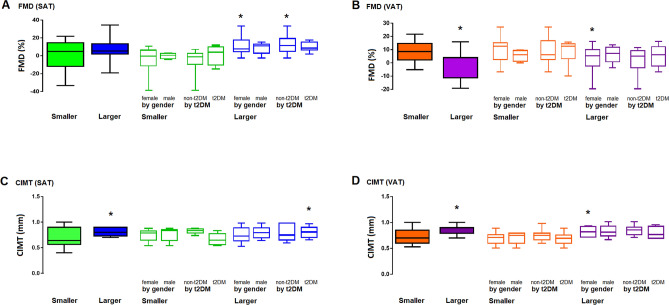
Cardiovascular risk. FMD (**A**,**B**) and CIMT (**C**,**D**) values arranged according to adipose tissue type. Results were further sub-arranged by gender and Diabetes Mellitus. SAT, subcutaneous adipose tissue; VAT, visceral adipose tissue; FMD, flow mediated dilation; CIMT, carotid intima media thickness. Figure was produced with the software Graph Pad Prism 7.0. URL https://graphpad-prism.software.informer.com/7.0.

Adipocyte size was related with surrogate indicators of subclinical vascular dysfunction and atherogenesis, such as FMD and CIMT (Fig. 4). In general, larger adipocytes were related with higher CIMT measures; whereas only VAT’s larger adipocytes were related with lower FMD values. Such relation of adipocyte size-CIMT was modified by gender (in VAT adipocytes) and t2DM (in SAT adipocytes). Likewise, gender affected the relation adipocyte size-FMD, independently from their source; whereas t2DM modified the relation adipocyte size-FMD only in SAT adipocytes.

### Adipocyte’s size/source and interactions of cardiometabolic risk factors

Finally, multivariable analysis was performed to further evaluate potential interactions related to adipocyte size. We found that larger adipocytes from VAT were significantly associated with adiponectin, only when interacting with insulin (OR 6.2, CI95% 1.6 to 24.2, p = 0.008; adiponectin median cutoff 26.09 pg/mL insulin median cutoff 24.55 mUI/mL) Likewise, larger adipocytes from SAT were associated with higher systolic pressure, only when accompanied by higher values of resistin and Hb_A1c_ (OR 4.6, CI95% 1.3 to 16.4, p = 0.019; systolic pressure median cutoff 124 mmHg, resistin median cutoff 7172 pg/mL Hb_A1c_ median cutoff 5.9%).

## Discussion

The main finding of the present study was that adipocyte characteristics of size and source showed differences regarding their impact on cardiometabolic and atherogenic risks in population with obesity.

In general, larger adipocytes were associated with higher blood pressure, glucose and CIMT, as well as lower concentration of insulin, cHDL and adiponectin; suggesting that the adipocyte size reliably reflected cardiometabolic risk. Further findings regarding the metabolic role of adipocyte size/source showed that although all the study population showed clinically significant insulin resistance, as assessed by HOMA-IR; larger adipocytes from SAT were associated with a worse gluco-metabolic profile, denoted by significantly higher plasma glucose, Hb_A1c_ and lower plasma insulin, as compared with larger adipocytes from VAT. A possible explanation may come from the allostatic hypothesis perspective^35^. Adipocytes from SAT showed lower expandability, as evaluated by the range of adipocyte size (Fig. 1, panel 1 and 2) as compared to VAT adipocyte range size (Fig. 1, panel 3 and 4); which would suppose that SAT adipocytes have a more limited ability for lipid storage, prompting to ectopic lipid accumulation in myocytes, hepatocytes and beta cells, then causing gluco- and lipo-toxic effects such as insulin resistance and apoptosis. Consistent with this notion, smaller adipocytes from VAT, probably owning lower expandability than larger adipocytes, showed higher levels of resistin, a pro-inflammatory adipokine. However, other studies have failed finding correlation between adipocyte size and visceral mass expansion, as in the case of epicardial thickness. This may be due to the heterogeneity of adipocytes size in VAT from different body sites, or dissimilarities regarding BMI and gender distribution between study populations^20,36^.

Other possible involved mechanisms explaining adipocyte size influence on metabolic risk include: (a) adipocyte size-related GLUT-4 membrane distribution, as well as the higher intracellular accumulation of lipids in larger adipocytes, which have been proposed to affect insulin resistance, independently from an inflammatory response^9,37^; (b) the relation of adipocyte size with the secretion of several adipokines, including IL-6, IL-8, leptin and TNFα, which own pro-inflammatory properties^7^; (c) adipocyte size and density may lead to relative degree of cell hypoxia, promoting the expression of hypoxia inducible factor-1α (HIF-1α) which regulates metabolically relevant proteins like glucose transporters, visfatin, leptin, MMPs, VEGFα and AGPTL-4^38^.

Regarding the effect over vascular function, our results indicate that adipocyte size is related to subclinical atherogenesis, since larger adipocytes were associated with higher CIMT. Consistently, adipose tissue mass had been previously described to modify the CIMT^39^; whereas, the amount of SAT has been associated with blood pressure in animal models, which is probably explained by the activation of adipocyte renin-angiotensin local system. Likewise, the increase in angiotensin II has been observed after blood flow modification in SAT, in population with obesity^40^. In the other hand, the source of the adipocyte seemed to play a key role, since only larger adipocytes from VAT were related with lower values of FMD; supporting the notion of a source-specific contribution of adipose tissue for the CVR, probably explained by their own characteristics of remodeling ability as well as the endocrine and pro-inflammatory nature^12,41,42^.

**Figure 5:**
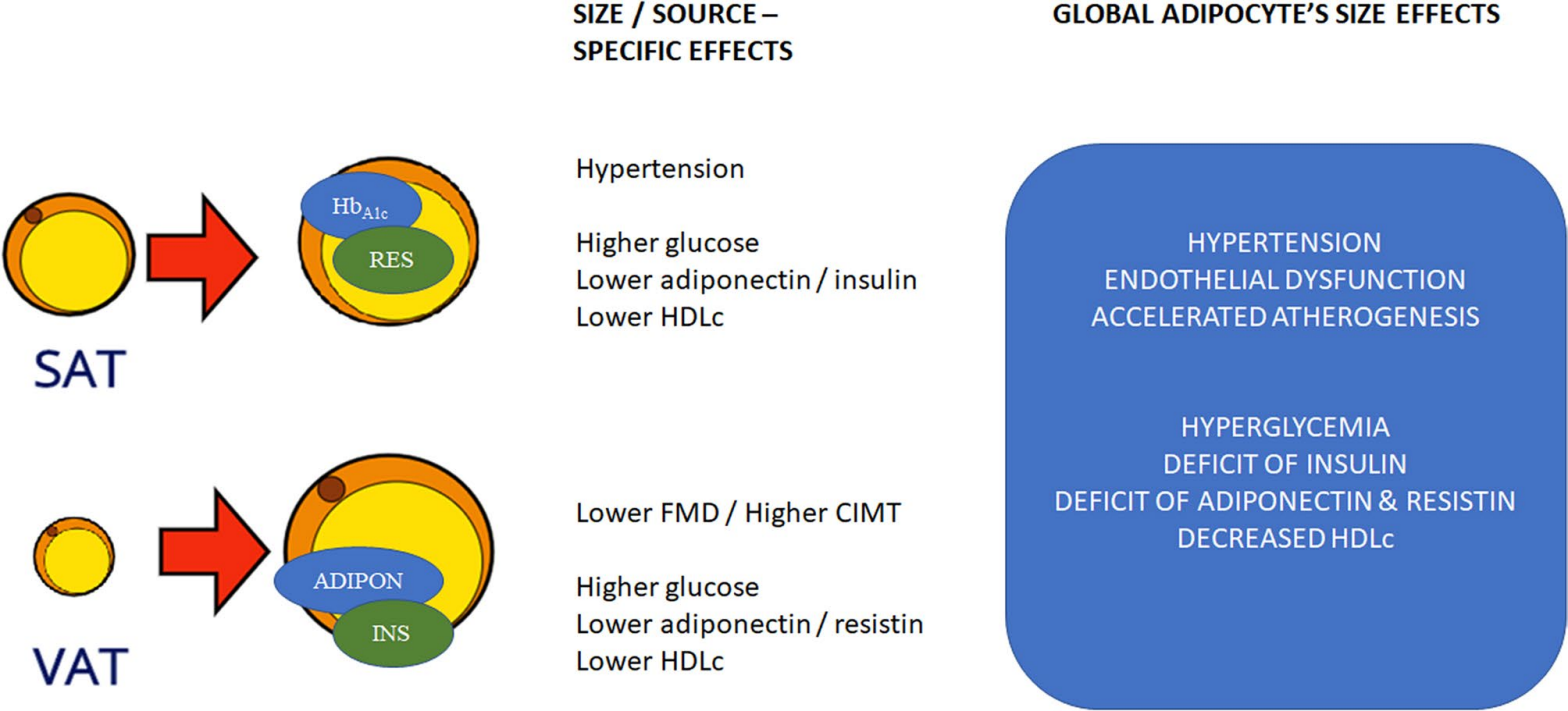
Adipocytes characteristics and cardiovascular risk. The diagram describes a proposed model, based on the results of adipocytes size/source, potential interactions with metabolic mediators and pathophysiological effects. SAT, subcutaneous adipose tissue; VAT, visceral adipose tissue; Hb_A1c_, glycated hemoglobin; RES, resistin; ADIPON, adiponectin; INS, insulin; HDLc; high density lipoprotein cholesterol; FMD, flow mediated dilation; CIMT, carotid intima media thickness.

Gender and t2DM significantly impacted the relation of adipocyte size/source with the metabolic and atherogenic risk. Gender showed to modify the effect of adipocyte size on several surrogate variables of atherogenic risk; whereas both, gender and t2DM affected the relation of adipocyte size/source on endothelial dysfunction and atherogenic markers. Furthermore, a multivariate analysis suggests that adiponectin-insulin interactions are related to larger adipocytes from VAT; as well as Hb_A1c_-resistin- systolic blood pressure interactions related to SATs larger adipocytes, probably reflecting selective mechanisms and/or metabolic pathways promoting cardiometabolic risk, as shown in the proposed model (Fig. 5).

Finally, adipose tissue expansion and abnormal cytokine profile found in obesity lead not only to higher CVR, but also carcinogenesis. Although not fully elucidated, insulin resistance, dysregulated adipokine profile and chronic inflammation have been proposed to increase cancer risk. Changes in lipid metabolism may promote cancer development by a free fatty acid increase, due to its functions as energy source for tumor cells and oncogenic signal^43,44^. Interestingly, we observed changes in lipid metabolism enzymes and lower plasma adiponectin associated to larger adipocytes, particularly affected by gender and t2DM in VAT, suggesting that higher VAT expansion and t2DM provide a higher susceptibility for cancer, particularly important in post-menopausal females due to the metabolic implications. This notion is consistent with findings from other studies^45–48^.

We consider that our data own reasonable external validation, since characteristics of the study population are comparable to other populations recruited in similar studies^39,41^. In addition, there is only a low number of studies characterizing adiposity, which have analyzed simultaneously SAT and VAT; whereas CVR is commonly estimated indirectly using atherogenic risk calculations based on lipids. The present study includes direct indicators of early vascular damage, namely FMD and CIMT.

Our findings may contribute to a better understanding of the link between adiposity characteristics and cardiometabolic risk; however, some limitations include: (a) the cross-sectional design, which may not reflect actual cardiovascular risk along time; (b) low sample size leading to potential bias, particularly in sub-analyses; (c) lack of desirable evaluations, such as determination of a higher number of adipokines and/or ectopic lipid accumulation.

In conclusion, adipocyte morphology and source differentially affect cardiometabolic and atherogenic risk in population with obesity, and are potentially influenced by gender and Diabetes Mellitus.

## Methods

### Design and study sample

Cross-sectional study, evaluating cardiometabolic phenotypes corresponding to adipocyte’s size and source. Additional subanalyses by gender and t2DM were performed. We enrolled patients > 18 years old, diagnosed with morbid obesity (Body Mass Index [BMI] higher than 40 kg/m^2^ or BMI higher than 35 kg/m^2^ experiencing obesity-related health conditions, such as t2DM, hypertension or obstructive sleep apnea/hypopnea), programmed for bariatric surgery between January 2016 and December 2017 at C.M.N. “20 de Noviembre”, ISSSTE at Mexico City. All females in the study sample were pre-menopausal. Patients did not received weight-reducing therapy during 6 months previous to the enrollment and they were excluded in case of second bariatric surgery, the existence of inflammatory diseases, severe renal and/or hepatic disease, active malignancy, pregnancy or evidence of history of cardiovascular disease, considered if self-reported or diagnostic evidence of ischemic heart disease, coronary artery disease, myocardial structural abnormalities, cardiac interventions or being under treatment for any of such conditions. The study was designed and performed according to ethical guidelines of the 1975 Declaration of Helsinki^49^, and approved by the Local Committees of Research, Ethics in Research and Biosafety of the Centro Médico Nacional ‘20 de Noviembre’ ISSSTE, Mexico City (Protocol ID No. 386.2013). All participants provided written informed consent.

### Clinical characterization

BMI was calculated as weight/height^2^^50^. Waist circumference was measured halfway between the lowest rib margin and the iliac crest at the end of a normal expiration. Blood pressure was obtained while the patient was in a seated position and was considered to be the mean of three readings obtained 5 min apart using an aneroid sphygmomanometer (Welch Allyn Inc.; Skaneateles Falls, NY, USA).

### Clinical biochemistry and other biomarkers

Following a 12 h starvation, venous blood samples (4 ml) were collected into BD Vacutainer tubes (Becton, Dickinson & Co., Franklin Lakes, NJ, USA) containing EDTA, and centrifuged at 1500*g*, 10 min, at 4 °C, separated into fractions and stored at − 80 °C prior to analyses. Plasma levels of triglycerides, total cholesterol, high-density lipoprotein cholesterol (HDLc), low-density lipoprotein cholesterol (LDLc), glycosylated hemoglobin (Hb_A1c_) and glucose were determined using routine clinical laboratory auto-analyser (Synchron CX9 PRO Clinical System; Beckman Coulter, Brea, CA, USA). HOMA-IR was calculated as follows: (Glucose [mg/dL] × Insulin)/405)^51^. Plasma TNFα, resistin and adiponectin were determined by immune-magnetic multiplexing assay (Milliplex MAP Human TH17 Magnetic Bead Panel Thermo Fisher Scientific USA, Milliplex MAP Adiponectin Magnetic Bead Panel Thermo Fisher Scientific USA, Milliplex MAP Resistin Magnetic Bead Panel Thermo Fisher Scientific USA) and read in Magpix System following recommendations of the provider.

### Adipose tissue analysis

During the bariatric surgery, samples of 2 cm^3^ of abdominal Subcutaneous Adipose Tissue (SAT) and Visceral Adipose Tissue (VAT) from the omentum, were obtained, considering all the aspects of ethics and biosafety of the patient. The size of the adipocytes was analyzed in histologic preparations stained with toluidine blue, which were further digitalized and measured with Image-J software (v. 1.53g. URL: https://imagej.nih.gov/ij). Data shown are the results of at least 10 fields performed on each slide.

### Endothelial dysfunction and subclinical atherogenesis

Flow Mediated Dilation (FMD) test was performed using ultrasound imaging with a Philips EPIQ7L12-3 Broadband Linear Array Transducer (Royal Philips) in B-mode according to the recommendations of the International Brachial Artery Reactivity Task Force^52^. Carotid Intima Media Thickness (CIMT) was evaluated using an ultrasound imaging 4.0 MHz probe (Philips, Saronno, Italy). Measurements were according to the 2004 Mannheim Consensus^53^. Briefly, with the patient in a supine position, primary transverse and longitudinal scanning of the common carotid artery was performed, while focusing on the posterior carotid wall at the beginning of the carotid bifurcation and the common carotid artery. CIMT was acquired at a distance of approximately 1 cm from the bifurcation of the common carotid artery and considered as the greatest distance between the lumen-intima interface and the media adventitia interface. The result was expressed as the mean of at least four measurements. In all cases, reproducible measures were validated using acceptable intraclass correlation coefficients (> 0.85) for inter-observer reliability.

### Statistical analyses

Normality of data distribution was performed with Kolmogórov-Smirnov test. Continuous variables were expressed as the media ± SD; qualitative data were shown as n (%). Two-tailed, U-Mann Whitney or non-paired T-test, as well as Fisher Exact Test, were used as appropriate. Pearson correlation coefficient was applied to determine the relationship between clinical-biochemical biomarkers and adipocyte size. Finally, multivariate regression model analysis was performed. All statistics were performed using software GraphPad Prism software (v.7) as well as SPSS (v.23 SPSS Inc., Chicago, IL, USA). Statistical significance was considered if *p* ≤ 0.05.

## Data Availability

The datasets generated and analyzed during the current study are not publicly available due to privacy policies of the hospital and patients information; but are available from the corresponding author on reasonable request.
